# Single- and two-photon imaging of human micrometastases and disseminated tumour cells with conjugates of nanobodies and quantum dots

**DOI:** 10.1038/s41598-018-22973-8

**Published:** 2018-03-15

**Authors:** Fernanda Ramos-Gomes, Julia Bode, Alyona Sukhanova, Svetlana V. Bozrova, Mara Saccomano, Miso Mitkovski, Julia Eva Krueger, Anja K. Wege, Walter Stuehmer, Pavel S. Samokhvalov, Daniel Baty, Patrick Chames, Igor Nabiev, Frauke Alves

**Affiliations:** 1 Translational Molecular Imaging Group, Max Planck Institute for Experimental Medicine, Göttingen, Germany; 2 Light Microscopy Facility, Max Planck Institute for Experimental Medicine, Göttingen, Germany; 3 Emeritus Optogenetic Group, Max Planck Institute for Experimental Medicine, Göttingen, Germany; 4 Laboratoire de Recherche en Nanosciences (LRN-EA4682), Université de Reims Champagne-Ardenne, 51100 Reims, France; 5 Laboratory of Nano-Bioengineering, National Research Nuclear University MEPhI (Moscow Engineering Physics Institute), 115409 Moscow, Russian Federation; 6 Department of Gynecology and Obstetrics, University Medical Center Regensburg, Regensburg, Germany; 7 Aix Marseille Univ, CNRS, INSERM, Institut Paoli-Calmettes, CRCM, Marseille, France; 8 Clinic of Haematology and Medical Oncology, Institute of Diagnostic and Interventional Radiology, University Medical Center, Göttingen, Germany

## Abstract

Early detection of malignant tumours and, especially, micrometastases and disseminated tumour cells is still a challenge. In order to implement highly sensitive diagnostic tools we demonstrate the use of nanoprobes engineered from nanobodies (single-domain antibodies, sdAbs) and fluorescent quantum dots (QDs) for single- and two-photon detection and imaging of human micrometastases and disseminated tumour cells in *ex vivo* biological samples of breast and pancreatic metastatic tumour mouse models expressing human epidermal growth factor receptor 2 (HER2) or carcinoembryonic antigen (CEA). By staining thin (5–10 µm) paraffin and thick (50 µm) agarose tissue sections, we detected HER2- and CEA-positive human tumour cells infiltrating the surrounding tissues or metastasizing to different organs, including the brain, testis, lung, liver, and lymph nodes. Compared to conventional fluorescently labelled antibodies the sdAb-HER2-QD and sdAb-CEA-QD nanoprobes are superior in detecting micrometastases in tissue sections by lower photobleaching and higher brightness of fluorescence signals ensuring much better discrimination of positive signals versus background. Very high two-photon absorption cross-sections of QDs and small size of the nanoprobes ensure efficient imaging of thick tissue sections unattainable with conventional fluorescent probes. The nanobody–QD probes will help to improve early cancer diagnosis and prognosis of progression by assessing metastasis.

## Introduction

Cancer still remains a great challenge to public health and represents a giant economic burden to the society. In 2013, more than one and a quarter million people died from cancer in the European Union^1^. To improve cancer prognosis and, hence, patients’ survival, early detection of the disease is one of the main purposes in diagnostic approaches. In this regard, the rapidly progressing field of nanotechnology is considered a powerful tool in cancer diagnostic and therapeutic applications^2,3^. The development of new nanoprobes which can detect cancer biomarkers with high specificity and sensitivity is appealing. The use of nanomaterials brings a number of advantages not only in diagnostics, but also in therapeutic concepts, such as improvement of water compatibility of hydrophobic drugs, their protection from degradation, with the result of a longer release, increase in drug bioavailability, tumour selectivity, and greater penetration depths for the treatment of deep seated tumours^4,5^. Among the enormous variety of nanoprobes available, semiconductor quantum dots (QDs) exhibit advanced optical properties and attract increasing attention for applications in biomedical research, such as cancer marker detection and tumour cell imaging^6^. QDs are a class of highly fluorescent homogeneous semiconductor nanocrystals 2–10 nm in size, composed of a photoluminescent semiconductor core coated with a shell of another semiconductor with a larger bandgap^7^. QDs have been shown to have broad absorption spectra, which allow excitation of QD of different sizes (fluorescence emission) at the same wavelength remote from their respective narrow emission bands, resulting in emissions of different fluorescence colors that can be detected simultaneously. Further advantages are a quantum yield close to 100%; high single-photon and two-photon molar extinction coefficients (orders of magnitude higher compared to organic dyes) and a high resistance to photobleaching and chemical degradation^8,9^.

To ensure cell specificity, QDs are normally bound to recognition molecules, such as antibodies, aptamers or peptides. Here, we used antibody fragments derived from llama antibodies, often called “nanobodies”, which are much smaller than conventionally used full-size IgG antibodies, with a molecular weight of only 13 kDa. These single-domain antibodies (sdAbs) are the smallest antibody fragments capable of binding their antigens with affinities comparable to conventional antibodies^6,10^. sdAbs are characterized by a low tendency to aggregate; they diffuse much better into tissues than full-size IgGs, and their size allows the labelling of thicker tissue segments than conventional IgGs do^11^. Because of these advantages, we have recently conjugated QDs to nanobodies in a highly oriented fashion, with all antigen binding sites facing outwards, which considerably increases the nanoprobe sensitivity for diagnostic and possible therapeutic use in oncology^12,13^ and demonstrated their advantages in breast and lung cancer cell imaging^14^.

Among the large number of tumour-associated antigens that can be targeted, HER2 receptor was found to be overexpressed on the cell membrane of various tumour types, including lung, ovarian, stomach, uterine, and especially breast cancers, where 25–30% of cells are HER2-positive. The expression level of this protein in breast cancer patients is mostly used to select patients for treatment with trastuzumab (Herceptin), a monoclonal antibody that recognizes and blocks the HER2 protein expressed on tumour cells^15,16^. Another glycoprotein most widely used as a tumour marker is CEA, whose level has been reported to be elevated in colorectal, breast, lung, pancreatic, liver, stomach, thyroid, prostate, bladder, and ovarian cancers^17^. CEA is often expressed in pancreatic ductal adenocarcinoma (PDAC), which accounts for over 90% of all pancreatic cancers, a devastating malignancy with extremely poor prognosis. PDAC normally grows fast, metastasizes early, and is generally accompanied by noticeable resistance to adjuvant therapeutic strategies^18^. In clinical oncology, CEA is commonly used as a serum marker as part of both preoperative staging and follow-up of the response of cancer patients to surgery and chemotherapy^19^.

Different metastatic mouse models of breast tumours have been described, such as the transplantation of BT474 and SKBR3 cell lines, which express high levels of HER2 receptor^20–23^ and MDA-MB-231 cells, which express low levels of HER2^24^. In the BT474 model, implantation of human breast tumour cells together with estradiol/norgestrel hormone pellets results in an increase in its metastatic potential^25^. Like these breast tumour models, PDAC mouse models are very invasive: AsPC1 and Capan-1 cells express high levels of CEA receptor, and, particularly, the AsPC1 model starts to develop symptoms as early as 4 to 5 weeks after implantation, with massive invasion in the neighbouring organs^18,26^. In this study we have shown that QDs conjugated in a highly oriented manner with the sdAbs targeting either HER2 or CEA are powerful tools for detecting human micrometastases and disseminated HER2- or CEA-positive tumour cells even in thick tissue sections of biological samples of metastatic breast and pancreatic tumour mouse models, respectively.

## Results

### Metastatic spread in human breast tumour mouse models

**Figure 1:**
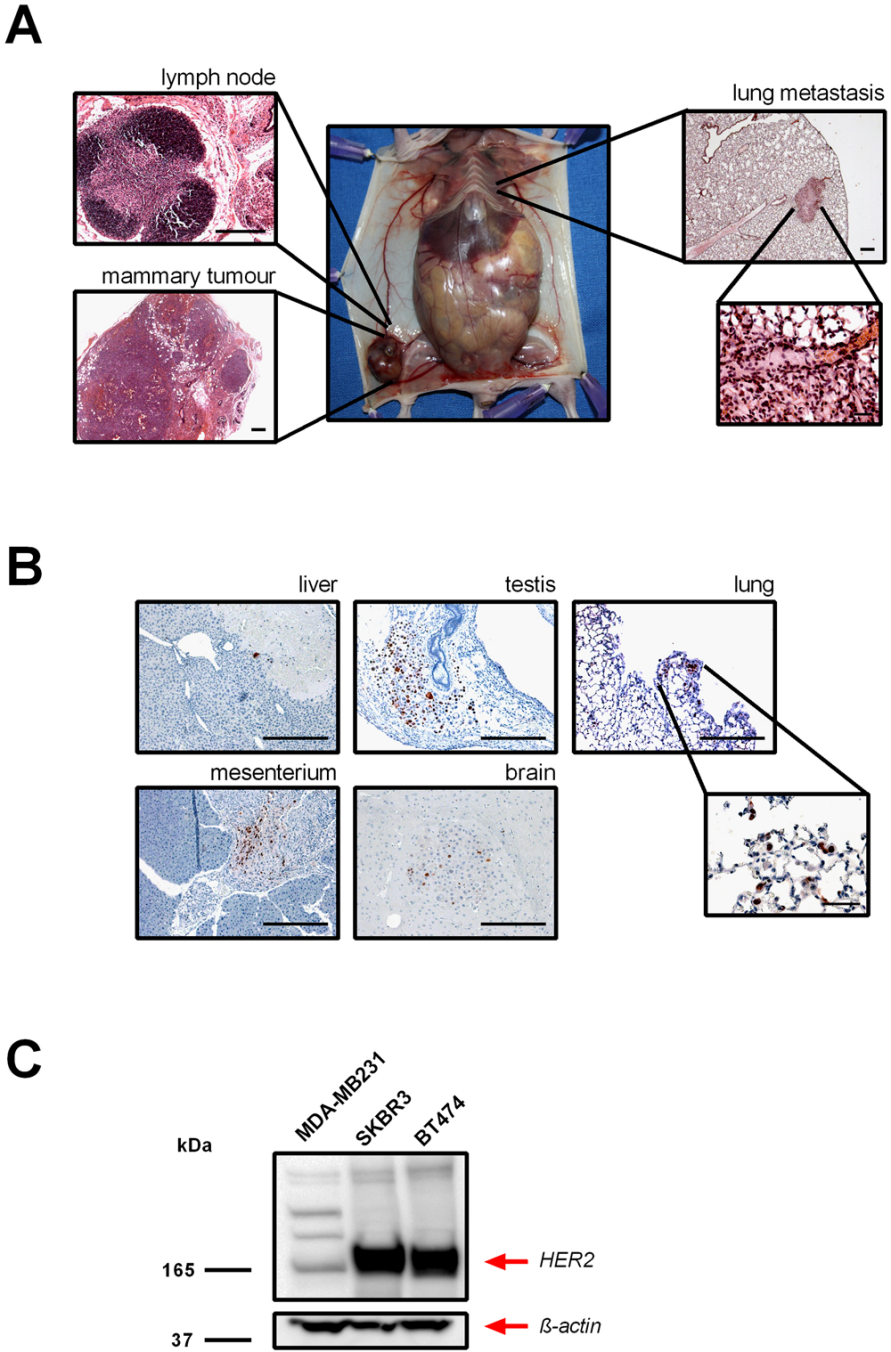
Macroscopic and histological appearance of the mouse breast tumour models. (**A**) Representative macroscopic images of the HER2-positive BT474 mouse model of human breast tumour are shown. Haematoxylin and eosin (H&E) staining shows a tumour-cell-infiltrated lymph node, a solid orthotopic breast tumour, and a lung metastasis. Scale bar, 500 µm. A higher magnification of the lung section shows the extravasation of tumour cells through a blood vessel. Scale bar, 50 µm. (**B**) Representative images of Ki-67-positive tumour cells (stained in brown) in samples obtained from the HER2-positive SKBR3 mouse model of human breast tumour. Both models resulted in metastatic spread of tumour cells in different organs, including the liver, testis, mesentery, brain, and lung. Scale bar, 500 µm. The inset shows a higher magnification of a section of the lung (scale bar, 100 µm). (**C**) Western blot analysis using anti-HER2 antibody (upper panel) shows the high expression of HER2 in human SKBR3 and BT474 cells used to produce the mouse breast tumour xenografts. A very low HER2 expression is shown in MDA-MB-231 cells used to obtain the HER2-negative control tumours. Staining of actin reveals comparable amounts of total protein in all samples (lower panel). The blots were cropped just for presentation purposes. Full-length gel graphs are presented in the supplementary information (Supplementary Figure S1).

Approximately 4–5 weeks after implantation of BT474 human breast tumour cells, primary tumours developed in the right inguinal mammary gland, accompanied by an enlargement of the inguinal lymph nodes (Fig. 1A). Representative haematoxylin and eosin (H&E) stained sections illustrate the primary tumour and the distorted architecture of the lymph nodes infiltrated by tumour cells. In particular, in lung tissue, tumour cells extravasated into the lung through a dilated blood vessel (Fig. 1A, inset). The second model, where immunosuppressed mice were first irradiated and then transplanted with human SKBR3 breast cancer cells directly into the liver, displayed disseminated tumour cells in different organs. Histological analyses of the liver, testis, mesentery, lung, and brain demonstrated massive infiltration of highly proliferating breast cancer cells, as validated by positively stained tumour cells with the Ki67 proliferation marker (Fig. 1B). As shown in Fig. 1C, Western blot analysis confirmed a high protein expression level of HER2 in BT474 and SKBR3 tumour cells and a low expression level in MDA-MB-231 tumour cell. An intense band of ~185 kDa, which corresponds to the expected weight of the HER2 protein, was detected in BT474 and SKBR3 cells lysates, while a faint signal was found in MDA-MB-231 cells used as a negative control (Fig. 1C).

### Binding specificity and sensitivity of sdAb-HER2-QD nanoprobes and pAb-HER2-Alexa Fluor

**Figure 2:**
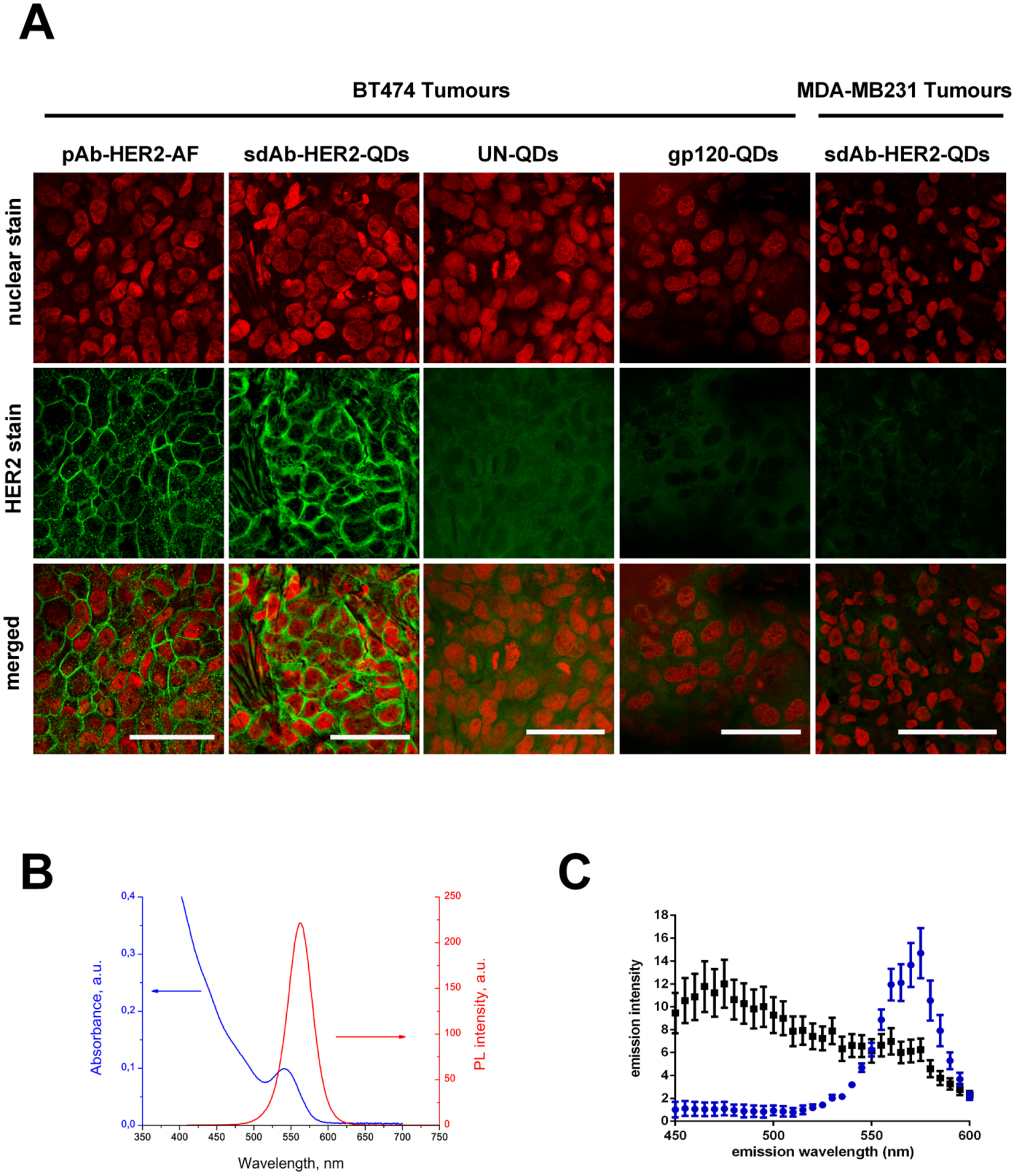
Immunofluorescence detection of HER2 protein in BT474 and MDA-MB-231 breast tumour sections using sdAb-HER2-QDs. (**A**) Signals produced by the binding of pAb-HER2-AF (green signal, *first column*), sdAb-HER2-QD (green signal, *second column*), UN-QD (*third column*), and gp120-QD (*forth column*) nanoprobes to tumour cells. Images were analysed by confocal microscopy (Leica SP5) with excitation at 405 nm. Signals derived from HER2-negative MDA-MB-231 tumours are shown (*right column*). Nuclear staining was performed with DRAQ5 (red signal, *upper row*) excited at 647 nm. Scale bar, 50 µm. (**B**) The absorption (*blue curve*) and emission (*red curve*) spectra of the QDs. (**C**) Fluorescence emission spectrum derived from the samples shown in Fig. 2A stained with sdAb-HER2-QDs (*blue curve*) emitting at 570 nm and with UN-QDs (*black curve*).

We determined the capability of sdAb-HER2-QD nanoprobes to bind to HER2 receptors expressed on tumour cells. The cross reactivity of the antibodies was tested and proved to not react against mouse tissue (Data not shown). Thin agarose sections (20 µm) of tumours expressing high and low HER2 levels, BT474 and MDA-MB-231, show HER2 in green fluorescence, stained with the anti-HER2 polyclonal antibody NeuC-18 (pAb-HER2) in combination either with a secondary antibody labelled by Alexa Fluor (AF)−546 (pAb-HER2-AF) or with sdAb-HER2-QDs nanoprobes (Fig. 2).

Confocal microscopy images (Fig. 2A) using either pAb-HER2-AF commercial antibody or sdAb-HER2-QDs show comparable high-intensity green signals at the cell membrane of BT474 tumour cells (Fig. 2A, *first and second columns*). When negative controls, i.e. unconjugated QDs (UN-QDs) or QD conjugated to irrelevant sdAbs (gp120-QDs) were applied onto thin agarose sections of high-HER2-expressing tumours, no comparable signals at the tumour cell membrane were detected (Fig. 2A, *third and fourth column*), which reflected the high specificity and sensitivity of sdAb-HER2-QDs for binding to HER2-expressing tumour cells. Although we detected autofluorescence on BT474 tumour cells (Fig. 2A, *third, fourth and last column*), this signal is negligible in comparison to the signal generated by the binding of the sdAb-HER2-QDs (Fig. 2A, *second column*). The staining of thin agarose sections of MDA-MB-231 tumours, which express low levels of HER2, with sdAb-HER2-QD nanoprobes did not result in any membrane stain either (Fig. 2A, *last column*).

The sdAb-HER2-QD nanoprobes are characterized by a broad absorption spectrum and a narrow emission peak (Fig. 2B). The results of spectral analysis of sdAb-HER2-QDs-stained in tumour samples are shown in Fig. 2C. In the samples stained with sdAb-HER2-QDs (Fig. 2C, *blue curve*) a fluorescence emission peak was detected at 570 nm. Instead, when the samples where stained with UN-QD nanoprobes, no specific signal of the QDs was detected on the tumour sample (Fig. 2C, *black curve*), thereby demonstrating the specificity of sdAb-HER2-QDs binding.

**Figure 3:**
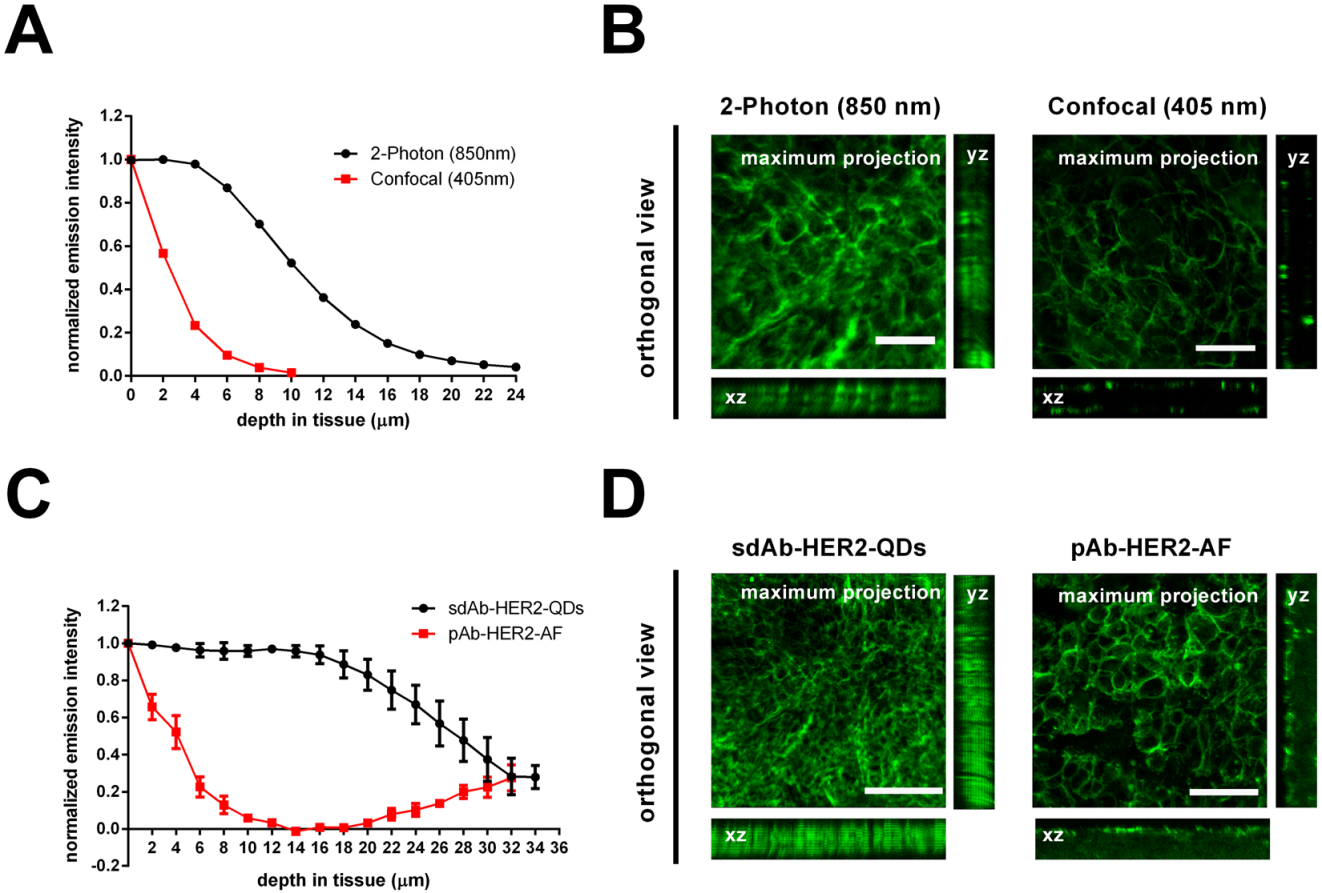
Deep tissue imaging of metastatic breast tumour samples using sdAb-HER2-QDs and pAb-HER2-AF. (**A**) Normalized fluorescence intensity of a BT474 breast tumour section (50 µm) stained with the sdAb-HER2-QDs and imaged with either 2P-LSM (•) or confocal microscopy (). (**B**) Orthogonal slices of z-stacks taken from tumour sections depicted in (A) demonstrate signal intensity throughout the section (x, z-view and y, z-view) and a maximum projection of the images from the 50 µm stack when imaged at the 2P-LSM or at the confocal microscopy. Scale bar, 25 µm. (**C**) Normalized fluorescence intensity of two high HER2 expressing breast tumour sections (80 µm), KPL-4 and BT474, respectively stained with pAb-HER2-AF () or sdAb-HER2-QDs (•). (**D**) Orthogonal slices of z-stacks taken from tumour sections depicted in (C) demonstrate signal intensity throughout the section (x, z-view and y, z-view) and a maximum projection of the images from the 40 µm stack when labelled with sdAb-HER2-QDs or with the pAb-HER2-AF. Scale bar, respectively 100 µm and 50 µm.

In order to evaluate the feasibility of using the sdAbs-QD conjugates in thick tissue sections of 50 µm and to explore the potential advantage of the small size of the nanoprobes for two-photon imaging of deeply seeded tumours, we embedded the tumours in agarose before immunostaining using the free-floating protocol. Figure 3A represents a graph showing the normalized emission intensities measured in the same tissue section using either two-photon laser scanning microscopy (2P-LSM) or single-photon confocal microscopy at 850 nm or 405 nm excitations, respectively. The normalized emitted fluorescence of sdAb-HER2-QDs was only reduced by up to ~50% in a tissue depth of 10 µm when imaged with 2P-LSM, while the use of a confocal microscope resulted in a ~98% reduction of fluorescence intensity. At the orthogonal views (Fig. 3B) we can see how light can deeper go into the tissue when imaged at the 2P-LSM when compared to the confocal excitation. Comparing the total emitted fluorescence at the 2P-LSM from the pAb-HER2-AF and the sdAb-HER2-QDs in deep tissue, we found that for the sdAb-HER2-QDs the emitted fluorescence was 2-fold stronger than the one emitted by the pAb-HER2-AF. The difference was not just at the maximum emitted fluorescence, but the fluorescence decay through the tissue was strongly improved by the use of the sdAb-HER2-QDs. At a depth of 10 µm the emitted fluorescence by the pAb-HER2-AF was almost abolished, with a reduction of up to ~90% of the initial fluorescence (Fig. 3C). Immunolabelling was performed in a free-floating protocol and labelling should be observed throughout the tissue section. As observed at the orthogonal views (Fig. 3D), while the pAb-HER2-AF sections show a poor labelling through the section, as the antibody permeability is impaired, we notice that for the sdAb-HER2-QDs sections this is not the case, which show labelling throughout the section. In the case of the pAb-HER2-AF we can even notice that at the end of the section the emitted fluorescence increases again (Fig. 3C) due the permeability of the antibody from the bottom side of the section from the free-floating solution. A maximum intensity projection of the z-stacks illustrates the quality of the images obtained when the sdAb-HER2-QDs were imaged at the 2P-LSM or at the confocal microscopy (Fig. 3B) or when we compared the emitted signal by the sdAb-HER2-QDs with the pAb-HER2-AF (Fig. 3D), both at the 2P-LSM.The results of these studies are illustrated by the videos V1 and V2 presented in Supplementary Information.

### Detection of micrometastases and disseminated tumour cells in breast tumour mouse models using sdAb-HER2-QDs

**Figure 4:**
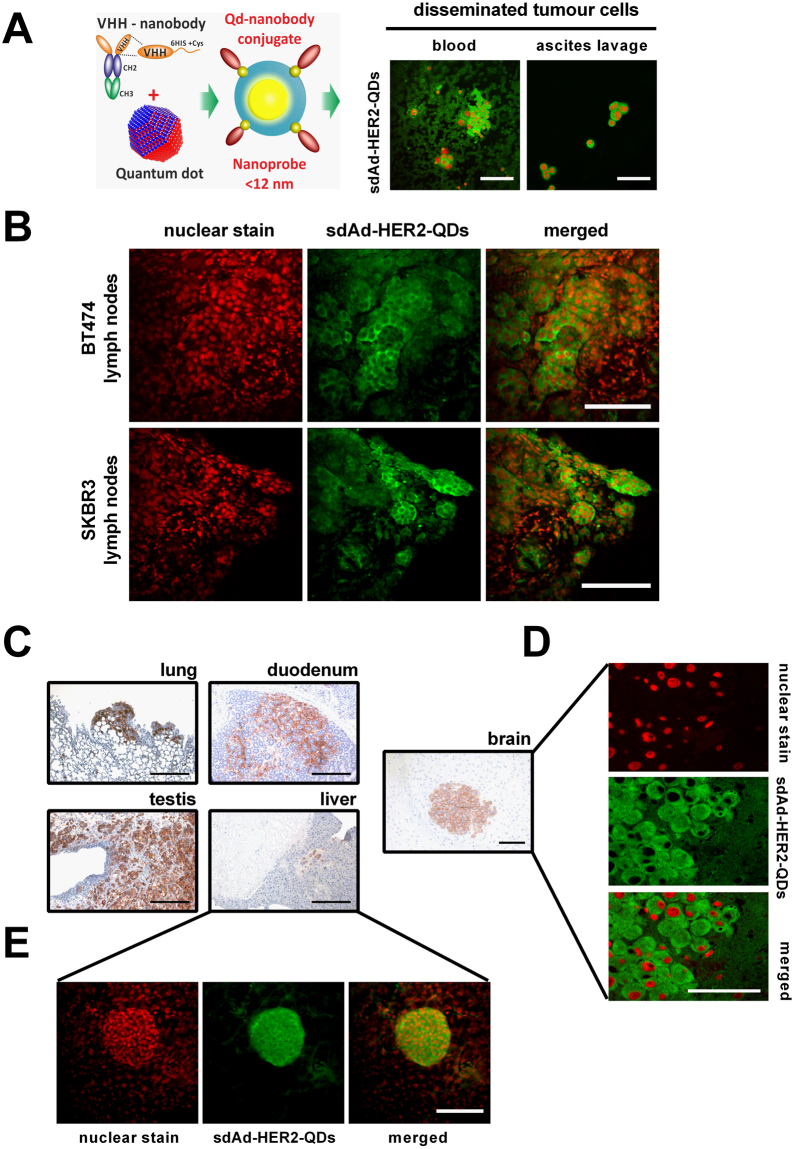
Detection of disseminated and metastatic tumour cells located in 50-µm sections of BT474 and SKBR3 breast tumours using sdAb-HER2-QDs. (**A**) Schematic representation of the QD nanoprobes and circulating tumour cells identified by sdAb-HER2-QD in fluorescent microscopy in blood and in ascites lavage. (**B**) 2P-LSM images showing the signals produced by sdAb-HER2-QD nanoprobes (green signal, *middle column*) in HER2-positive tumour cells in 50-µm agarose sections of lymph nodes from two mouse models, BT474 and SKBR3. Nuclear staining was performed with Hoechst (red signal, *left column*). Scale bar, 100 µm. (**C**) Microscopic images of HER2-positive cells identified in the lung, duodenum, testis, liver (scale bar, 500 µm), and brain (scale bar, 200 µm) of the SKBR3 breast tumour mouse model using mAb-HER2. The same cells identified with sdAb-HER2-QDs (**D**) in the brain by confocal microscopy and (**E**) in 50-µm liver sections by 2P-LSM.

Figure 4A illustrates a schematic diagram showing the sdAb linked to the QD via a Cys residue, forming a nanoprobe with the size <12 nm. Circulating SKBR3 breast tumour cells were detected in peripheral blood on blood smears and in metastatic cells from ascites lavage obtained from BT474 breast tumour mice. We observed the formation of clusters of SKBR3 cells or individual cells in blood samples and in ascites lavage, respectively, identified positively by sdAb-HER2-QDs (Fig. 4A, *right column*).

To investigate whether sdAb-HER2-QDs could detect small metastases with high signal intensity and high specificity in deep tissue, we used 50-µm agarose slices of lymph nodes infiltrated by tumour cells from the BT474 and SKBR3 human breast tumour mouse models. In Fig. 4B, inguinal lymph nodes composed of immune cells and tumour cells are shown, but only BT474 and SKBR3 tumour cells were specifically stained with sdAb-HER2-QDs (shown in green). One of the advantages of thick agarose slices over thin paraffin sections or cryosections is an improved preservation of tissue structures, to detect small metastases, by 2P-LSM after staining with the sdAbs-HER2-QD nanoprobes (Fig. 4B) within the lymph node.

The capacity of sdAb-HER2-QDs to specifically detect disseminated tumour cells was not limited to lymph node metastases, but was also shown for metastases in the liver, duodenum, lung, testis, and brain in the SKBR3 xenograft tumour mouse models (Fig. 4C–E). Immunohistochemistry of thin paraffin sections with conventional mAb-HER2 shows the locations of metastases in the examined organs, supporting the finding that sdAb-HER2-QDs specifically bind disseminated tumour cells (Fig. 4B–D). As can be seen from the immunofluorescence results, HER2-positive cells were also visualized in paraffin sections of the brain (Fig. 4D) and liver (Fig. 4E), demonstrating that sdAb-HER2-QDs have a high potential as imaging probes for the detection of metastatic HER2-expressing breast tumour cells in 50-µm tissue sections.

### Detection of tumour cells in orthotopic metastatic PDAC mouse models using sdAb-CEA-QDs

**Figure 5:**
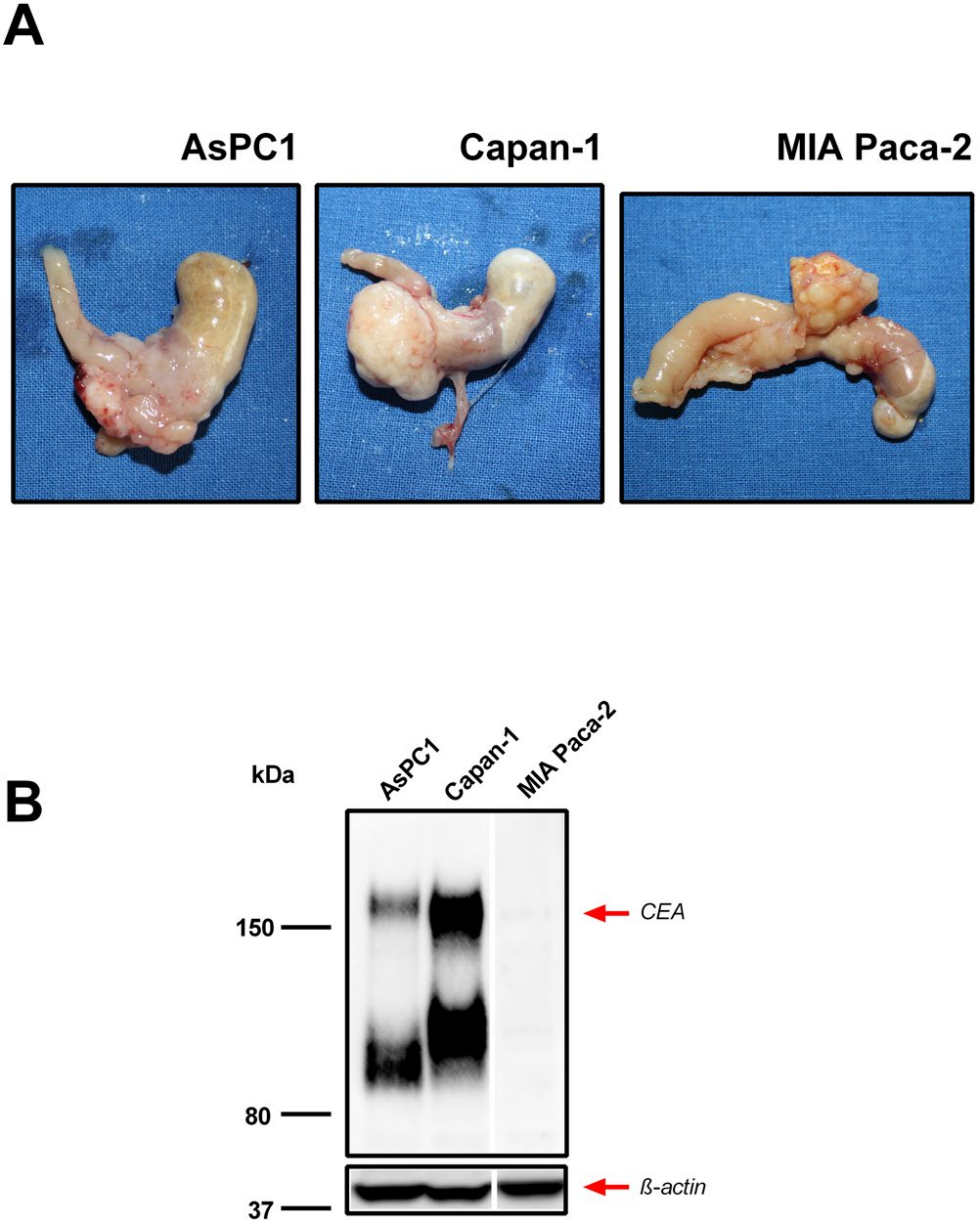
Macroscopic appearance of metastatic PDAC mouse models. (**A**) Representative images of high-CEA-expressing AsPC1 and Capan-1 and the low-CEA-expressing MIA Paca-2 primary PDAC tumours invading the neighbouring stomach and duodenum that developed in mice after orthotopic cell transplantation. (**B**) Western blot analysis using anti-CEA antibody (upper panel) detected a high expression of CEA in AsPC1 and Capan-1 cells, which were used to produce the xenograft PDAC mouse tumours. Only low CEA expression is observed in the MIA Paca-2 cells used to obtain CEA-negative tumours as controls. Staining of actin reveals comparable amounts of total protein in all samples (lower panel). The blots were cropped just for presentation purposes. Full-length gel graphs are presented in the supplementary information (Supplementary Figure S2).

To evaluate the capacity of sdAb-CEA-QDs to detect CEA-positive human tumour cells invading different mouse tissues, we obtained biological samples from three metastatic PDAC mouse models orthotopically transplanted. Figure 5A shows the macroscopic appearance of different primary PDAC tumours and their characteristic invasion into the stomach and duodenum after orthotopic cell implantation. The CEA expression pattern of PDAC cells used to generate different xenograft tumour mouse models was verified by Western blot analysis (Fig. 5B). A strong band at ~180 kDa, which is comparable with the expected weight of the CEA protein, was detected in AsPC1 and Capan-1 cell lysates, while no protein band was found in MIA Paca-2 cell lysates used as low-CEA-expressing control cells.

**Figure 6:**
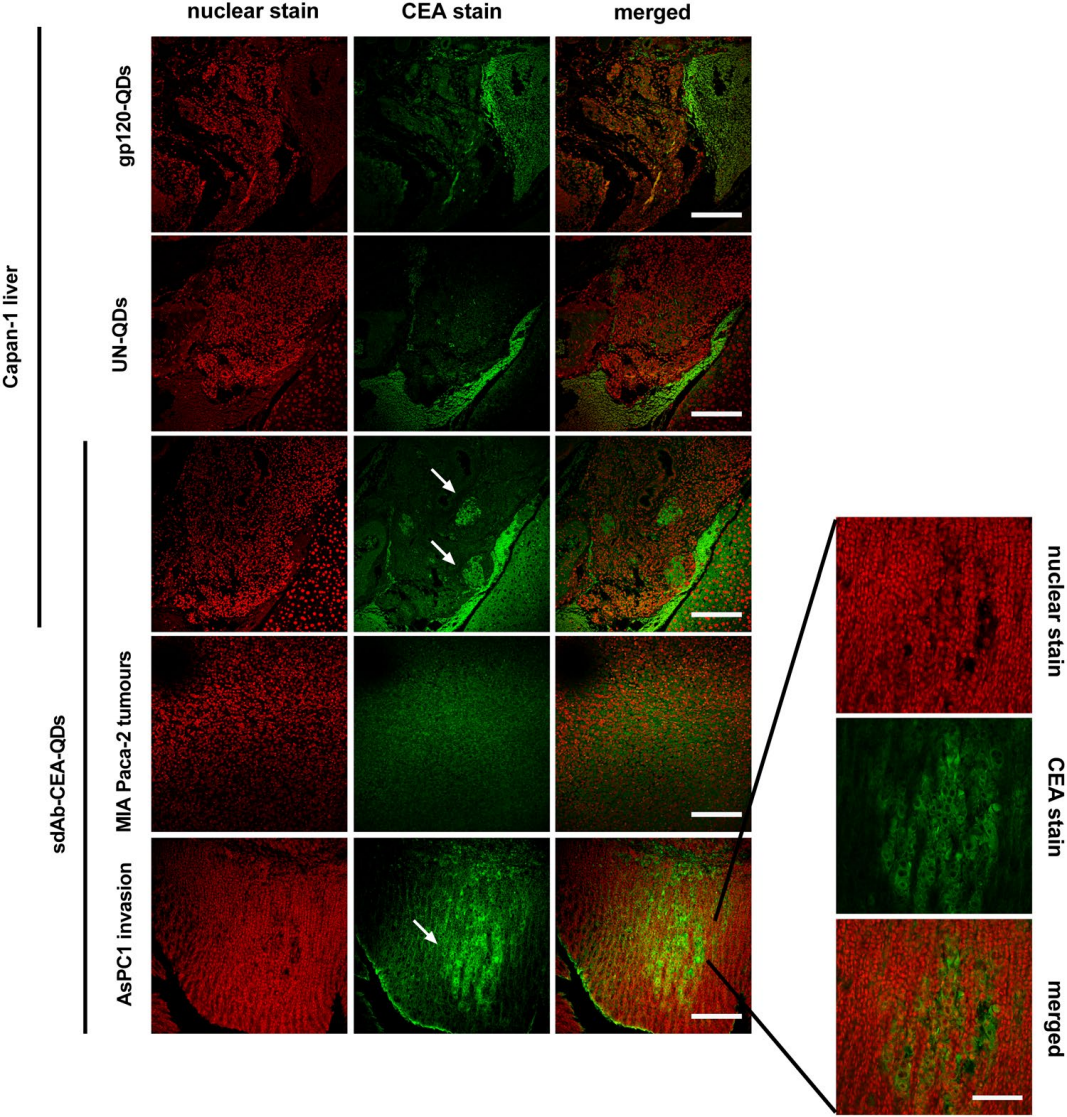
Detection of CEA expressing cells in sections of PDAC using sdAb-CEA-QDs. Confocal images showing the signal produced by sdAb-CEA-QD nanoprobes (green signal) in thin paraffin sections of PDAC tumours expressing CEA. The invasion of CEA-positive tumour cells is seen in intestinal crypts (in AsPC1 tumours, *last row* and in higher magnification, scale bar 100 µm) and in the liver (in Capan-1 tumours, *third row*). The control nanoprobes were analysed using high-CEA-expressing Capan-1 liver sections. UN-QD (*second row*) and gp120-QD nanoprobes (*first row*) detected no positive signal for CEA expression. The negligible signal produced by CEA-negative MIA Paca-2 tumours is also shown (*forth row*). Nuclear staining was performed with DRAQ5 (red signal, *first column*). Scale bar, 200 µm.

In paraffin tissue sections of the AsPC1 tumour mouse model, the invasion front consisting of tumour cells growing into the intestinal crypts was seen as intense green fluorescence signals, due to the specific binding of the sdAb-CEA-QD nanoprobes to human tumour cells (Fig. 6, *first column* and higher magnification picture). A small group of metastases was also detected as bright green signals by confocal microscopy in the liver of the Capan-1 tumour mouse model stained with sdAb-CEA-QDs (Fig. 6, *third column, arrow*). In control tissue sections from MIA Paca-2 tumours with low expression levels of the CEA antigen, no positive staining with sdAb-CEA-QDs was detected (Fig. 6, *second column*). The use of the negative control nanoprobes, UN-QDs and gp120-QDs, on thin paraffin sections of the high-CEA-expressing Capan-1 tumour yielded no positive signal, thereby confirming that QDs do not unspecifically bind to tumour cells (Fig. 6, *last two columns*).

## Discussion

Our study shows that QD-labelled sdAbs targeting the tumour-associated antigen HER2 or CEA are highly efficient tools for the detection of *not only primary tumours, but also disseminated tumour cells and micrometastases in different organs with high brightness and sensitivity*. We also provide evidence demonstrating that sdAb-QDs nanoprobes are superior over classical ones, such as Alexa Fluor, for example. In particular, we demonstrate a specific binding of sdAb-HER2-QDs to HER2 expressed on mammary tumour tissue of SKBR3 and BT474 mice, with the emission of more intense signals compared to a commercial antibody labelled with AF (pAb-HER2-AF). Characterized by a diameter below 12 nm, these nanoprobes are composed of a QD conjugated with four homogeneously oriented copies of sdAbs^12,13^. These highly oriented sdAb-QD nanoprobes have already been shown to have a very broad absorption band, with the highest extinction in the UV spectral region, contrasting with have the narrow absorption range of Alexa fluorophores of about 130 nm^14^. Although the absorption band of QDs is very broad, their emitted fluorescence can be tuned with high precision by varying the size and composition of the fluorescent core. Thus, when the core of QDs is composed of CdSe, a variation of core size in the 2 to 8 nm range gradually shifts the emission peak of such QDs from 480 to 660 nm^27,28^. This constitutes a clear advantage of the QDs over Alexa fluorophores when used in multiplexed applications, since detection of different antigens in the same sample by using QD-based nanoprobes is possible with the use of a single light source. Like other organic fluorophores, the pAb-HER2 and AF546-labelled secondary antibody combination used in this study would be poorly suitable for multicolour signalling at a single wavelength. In contrast, all sdAb-HER2-QD nanoprobes are excited at 405 nm, and the emitted fluorescence is tuneable by varying their core size (in this study, its peak was located at 570 nm), making them emitting at different wavelengths of the spectrum^12^.

Our results also show that imaging of QDs using 2P-LSM results in a much slower attenuation of the fluorescence emission intensity when moving deeper into tissue as compared with the single-photon confocal microscopy, allowing the detection of signals from sources located farther from the tissue surface. This can be explained by one of the advantages of excitation with 2P-LSM over the excitation with confocal microscopy, namely, nonlinear excitation, which generates fluorescence emission proportional to the square of illumination intensity^6^.The 2P-LSM excitation is restricted to the focal plane, which entirely eliminates the out-of-focus signal, resulting in an increased resolution and less photobleaching. The excitation is performed using much longer wavelengths (700–1000 nm) which is known to penetrate tissue more deeply causing less light scattering and better signal-to-noise ratio. In our samples, we observed less photobleaching when using sdAb-HER2-QD nanoprobes compared with pAb-HER2-AF. We believe that this is related to the QD capability of producing stronger emission, in combination with their higher photostability due to the inorganic nature of the QD fluorophore. We have also shown that the use of sdAb-HER2-QDs is highly efficient in detecting circulating tumour cells and metastatic spread in lymph nodes of two xenograft breast tumour models. In both lymph nodes, we were able to clearly distinguish between HER2-positive tumour cells, which were specifically stained by the nanoprobes, and unstained leucocytes, which did not express the antigens. Strikingly, the 50-µm sections obtained from agarose-embedded tumours ensured a better preservation of the morphological structures of the tissue and higher quality of the fluorescent signals compared to thinner paraffin slices. SdAb-HER2-QDs have a higher diffusibility in thicker sections compared to the commercial pAb-HER2-AF, which makes them excellent probes for deep tissue imaging.

In the tumour samples studied, the nanoprobe fluorescence was reduced by ~50% of the initial emitted fluorescence at a depth of 10 µm when imaged by 2P-LSM. For comparison, the fluorescence emitted by pAb-HER2-AF was completely abolished at this depth. This is accounted by the difference in fluorescence decay and light scattering through the thick tissue section. The ability of the antibody to penetrate the tumour tissue section was probably impaired by the size and structure of the antibody and the complex composition a tumor presents. Many clearing methods, which improve imaging of the tissue allowing better light penetrance, exist and have often been used, however there are still not stablished protocols for clearing tumours tissues and which would not interfere with the architecture of the extracellular matrix. As we were interested in preserving such structures for later analysis of tumour cell - extracellular matrix interactions, such techniques were not desirable for our purposes.

As previously reported by Rakovich *et al*. (2014), the lifetime of sdAbs-QD nanoprobes is about 24 ns (a typical value for CdSe-based QDs). This is significantly higher than that of mAb-HER2-AF, which has been found to be about 3–4 ns, similar to the lifetime of the autofluorescence of cellular components, such as nicotinamide adenine dinucleotide (NADPH), with a fluorescence lifetime of approximately 2 ns. Thus, QD-based nanoprobes can ensure better sensitivity when used in combination with imaging techniques^14,29^. This characteristic is essential for the analysis of tumour tissues, which display alterations in the concentration and constitution of extra- and intracellular components, such as collagen, elastin, keratin, haemoglobin, and NADPH, leading in some cases to substantial autofluorescence^30^.

In this study, we demonstrated that sdAb-CEA-QDs specifically detect tumour cells in tissues of metastatic models of PDAC tumours. The invasion of tumour cells into healthy organs/tissues surrounding the primary tumour is reliably detected by their labelling with the developed nanoprobes. Overall, the small size of the nanoprobes is an advantage over full-size antibodies, allowing a higher diffusion of the probes through tissues, and their retention on tumour cells within a healthy tissue.

## Conclusions

We have demonstrated that sdAb-QD conjugates are effective tools to detect disseminated human tumour cells and micrometastases by binding HER2 and CEA, two antigens overexpressed in metastatic breast and pancreatic tumours, respectively. SdAb-QDs are shown to be largely superior to commercial polyclonal antibodies conjugated to conventional Alexa dyes. The 2P-LSM technique is shown to have considerable advantages over single-photon confocal microscopy to visualize the nanoprobes on metastatic tumours, since the emitted fluorescence generated by 2P-LSM is higher, resulting in a much better signal-to-background ratio. Additionally, the diffusibility of sdAb-QD nanoprobes through tissue facilitates the access to small metastases and complex structures, making these nanoprobes powerful tools for cancer diagnostic and therapeutic applications.

## Methods

### Cell Lines

Human PDAC cells AsPC1 (CRL-1682), Capan-1 (HTB-79), MIA Paca-2 (CRL-1420) and the human breast cancer cells BT474 (HTB-20), SKBR-3 (HTB-30), and MDA-MB-231 (HTB-26) were purchased from ATCC (Rockville, MD). KPL-4 cells were provided by J. Kurebayashi (Kawasaki Medical School, Kurashiki, Japan). BT474 and AsPC1 tumour cells were cultured in RPMI 1640 medium with 10% foetal bovine serum (FBS, Gold, PAA Laboratories Gold); MDA-MB-231 cells, in DMEM medium with 10% FBS; SKBR3 cells, in McCoy’s 5 A medium with 10% FBS; Capan-1, in IMDM medium with 20% FBS; and MIA Paca-2, in DMEM/F12 medium with 10% FBS and 2.5% horse serum (Biochrom). All media were from Life Technologies. All cells were maintained at 37 °C in a humidified atmosphere with 5% CO_2_, were harvested at ~80% confluency by brief trypsinization in a 0.05% trypsin–EDTA solution, washed several times, and placed into sterile PBS shortly before implantation.

### Conjugates of single-domain antibodies (sdAbs) and quantum dots (QDs)

Highly fluorescent QDs emitting at 570 nm were conjugated to sdAbs targeting HER2 and CEA, or to the protein gp120 through an engineered additional C-terminal cysteine residue. The protocols for sdAb development and isolation, QD synthesis, solubilisation in water, stabilization, and conjugation with sdAbs were described previously^13,31–34^.

### Animals and tumour cell transplantation

Animals were housed in individually ventilated cages and allowed food and water *ad libitum*. For tumour cell implantation 6–12 weeks old nude NMRI-*Foxn1*^*nu*^ mice (Charles River Laboratories) were anesthetized by intraperitoneal (i.p.) injection of 15 mg/kg xylazine (Ecuphar) and 75 mg/kg ketamine (Medistar) as described previously^15,35^.

For the BT474 mouse model, two hormone pellets were implanted at the neck of female athymic nude mice, each pellet containing 17β-estradiol and norgestrel (1.7 mg and 10 mg, respectively). Subsequently, 1 × 10^6^ BT474 cells resuspended in 20 μL of PBS or 5 × 10^6^ KPL-4 cells resuspended in 30 μL of PBS were orthotopically transplanted into the right abdominal mammary fat pad. For negative controls, 1 × 10^6^ low-HER2-expressing MDA-MB-231 cells were implanted orthotopically to generate xenograft tumours with a low HER2 expression.

The second model used was the high-HER2-expressing SKBR3 xenograft model, using NOD-*scid IL2Rγ*^null^ (NSG) mice obtained from Jackson Laboratories as described earlier (Wege *et al*., 2011). The animals were bred and kept in a specialized pathogen-free facility at the University of Regensburg. For obtaining xenograft mouse models, the animals were irradiated (1 Gy) and, 3 h later, transplanted with 3 × 10^6^ SKBR3 tumour cells. In all experiments, cells were transplanted into the liver of new-born mice. The mice were euthanized and analysed at the age of 3–5 months after transplantation^23^.

10–20 weeks old male athymic nude NMRI-*Foxn1*^*nu*^ mice (Charles River Laboratories), were orthotopically transplanted into the head of the pancreas with 1 × 10^6^ AsPC-1 or Capan-1, high-CEA-expressing human PDAC cells or negative controls, MIA Paca-2 low-CEA-expressing, all resuspended in 20 μL of PBS as described before^18^.

For analgesia, the mice received Rimadyl (carprofen; 5 mg/kg, Norbrook) i.p. during surgery and Metamizole (1.33 mg/ml, Zentiva) in drinking water for two days before and for three days after transplantation. Mice were examined three times a week for weight loss, general condition, and palpable tumour formation. After euthanization by CO_2_ asphyxiation and cervical dislocation mice were dissected and tumour sizes, numbers and locations of macroscopic metastases were assessed. All animal experiments were performed in accordance with German animal ethics regulations and were approved by the local ethics office of Lower Saxony (license nos. 33.9-42502-04-13/1085, 33.12-42502-04-13/1222, 33.14-42502-04-11/0656, 33.14-42502-04-10/0064 and 54-2532.1-27/11).

Blood was obtained by cardiac puncture from mouse with a heparin filled syringe. To obtain the cells from the ascites, several washes were performed at the abdomen cavity and blood and cells from ascites were prepared as smear slides.

### Protein extraction and separation and western blot analysis

To obtain cell lysates, cultures were washed twice with PBS, incubated in 3 mL of lysis buffer (50 mM Tris-HCl (pH 7.4), 300 mM NaCl, 5 mM EDTA, and 1% Triton X-100) containing a protease inhibitor cocktail (Roche) for 30 min and centrifuged for 15 min at 21,300 *g*. The supernatant was used as the total cell extract. The protein concentration was determined using BCA Protein Assay Reagent (Pierce).

Samples of 30 µg of total protein extracts reduced by 50 mM of sample reducing agent (RSA, Novex), were separated by SDS-PAGE (NuPAGE Novex TRIS acetate 3–8% gel, Invitrogen) and transferred to nitrocellulose membranes (Amersham Biosciences). The membranes were blocked with 5% bovine serum albumin (BSA, Sigma-Aldrich) in 0.05% TBS-T (Tris-buffered saline supplemented with Tween-20) for 1 h at room temperature (RT), probed for 2 h with either an anti-HER2 rabbit polyclonal antibody (pAb-HER2, Neu (C-18):sc-284, Santa Cruz Biotechnology, dilution of 1:200) or a an anti-CEA rabbit monoclonal antibody (mAb-CEA, EPCEAR7 ab133633, Abcam, dilution of 1:1000), each diluted in blocking solution. Mouse monoclonal anti-β-actin antibody was used as protein loading control (mAb-β-actin, Abcam 8226, dilution of 1:1000). Blots were incubated with a horseradish peroxidase (HRP)-conjugated anti-rabbit antibody (1:8000, GE Healthcare Life Sciences) and developed using chemiluminescent HRP substrate (Millipore Immobilon system). Signals were detected by using a Bio-Rad Chem-Doc luminescence detection system.

### Immunohistochemistry (IHC)

Tumour tissue and organs from all xenograft models were harvested and fixed overnight (ON) in 10% formalin at 4 °C. After that, the tissues were kept at 4 °C in a PBS containing 0.1% of sodium azide for preservation. The samples were embedded either in paraffin or in 5% agarose blocks for preparation of serial sections. From all models, 5-μm paraffin tissue sections were prepared, stained with haematoxylin and eosin (H&E), and examined by routine light microscopy. For IHC, 5-μm paraffin sections were de-paraffinized in xylene, rehydrated through ethanol (100, 96, 70 and 50%), and finally placed in TBS. Antigen retrieval was performed in a citrate buffer (2% citric acid and 8% sodium citrate) for 60 min at 95 °C and then 20 min at RT. The slices were incubated with 3% hydrogen peroxide inhibitor (DAKO) to quench the endogenous peroxidases and washed in TBS. Nonspecific binding was blocked by incubating the sections for 1 h in Sea block solution (ThermoScientific). For tissue IHC, either anti-Ki-67 rabbit monoclonal antibody (SP6, Cell Marque 1:500) or anti-HER2 rabbit monoclonal antibody (HER2/ErbB2 Epitomics, mAb-HER2, 1:100) was used ON at 4 °C. The slices were washed two times for 5 min in a Tris-buffer solution and incubated with secondary HRP-conjugated anti-rabbit antibody (Nichirei) for 1 h; the signals were developed with 3-amino-9-ethylcarbazole (AEC). The slides were counterstained with haematoxylin and mounted with Aqua Tex (Merck).

IHC with anti-HER2- or anti-CEA-sdAb-QDs (15 nM) conjugated nanoprobes were performed for 1 h in a humidified chamber at RT. Tumour tissues as negative controls were stained with a solution of either unconjugated (UN)-QDs or QDs conjugated to gp120 protein (gp120-QDs) (gp120 is an envelope glycoprotein exposed on the surface of the human immunodeficiency virus, HIV). For all fluorescent conjugates, the slices were washed three times in TBS before nuclear staining was performed with DRAQ5 (Biostatus, dilution of 1:1000), and sections were mounted using Cytoseal (Thermo Fisher Scientific).

Agarose tissue sections (30–50 μm) were obtained with a Vibratome (Leica VT1000S) and washed three times in TBS-T. All incubations were performed free-floating in solution, first with 3% hydrogen peroxide inhibitor (DAKO) followed by a washing step with TBS-T before. Nonspecific binding was blocked by incubation for 1 h in 2% BSA in TBS with 0.5% Tween (TBS-T). For tissue labelling, the agarose tissue sections were incubated with either anti-HER2 polyclonal antibody (pAb) (Neu (C-18):sc-284, Santa Cruz Biotechnology, 1:200) or anti-HER2 monoclonal antibody (Epitomics, 1:100) and then with Alexa Fluor 546 (Thermo Fisher Scientific) or anti-HER2-QDs (15 nM) for 2 h. Negative controls were stained with a solution of either UN-QDs or gp120-QDs (15 nM). Nuclear staining was performed with DRAQ5 (Biostatus, 1:1000), and tissue sections were mounted using Cytoseal (Thermo Fisher Scientific).

### Confocal and two-photon microscopies

First, a Leica SP5 confocal laser scanning microscope (CLSM, Leica Microsystems) equipped with hybrid detectors, a 40× immersion objective lens (HCX PL APO lambda blue), a motorized stage, and a tuneable laser (470–670 nm) was used. QDs, Alexa Fluor 546, and DRAQ5 were excited at 405, 561, and 647 nm, respectively, and their individual emissions were collected at 560–580, 560–620, and 665–795 nm. Second, a custom-made two-photon laser scanning microscope (2P-LSM) equipped with a femtosecond-pulsed titanium–sapphire laser (Chameleon Ultra II; Coherent) and a Zeiss W Plan Apochromat 20× (NA 1.0) water immersion objective lens was used. For excitation of the QDs, Hoechst and Alexa 546 the laser was respectively set at 850 nm and at 830 nm. The emitted light was split by two longpass dichroic mirror (Semrock) one 560 nm, and another 405 nm, and collected by photo-multiplier tubes (Hamamatsu). For the z-stacks acquisitions no laser power depth compensation was performed. All images were processed with the FIJI (an image-processing package based on ImageJ), Imaris 8.30 (Bitplane), Graph Pad Prism 6 (Graph Pad Software, Inc.) and Matlab (version 7, MathWorks) software. For 3D imaging of microscopy z-stacks as volumes, the Java-based ImageJ 3D Viewer plugin developed by Benjamin Schmid (Biozentrum Universität Würzburg) was used.

### Data availability

The datasets generated during and/or analysed during the current study are available from the corresponding author on reasonable request.

## Electronic supplementary material

Supplementary Information

Supplementary V1

Supplementary V2
