# Supplementary material for: Single- and two-photon imaging of human micrometastases and disseminated tumour cells with conjugates of nanobodies and quantum dots
Source: Sci Rep. 2018 Mar 15;8:4595. doi: 10.1038/s41598-018-22973-8 (PMC5854706; doi:10.1038/s41598-018-22973-8)
Supplement: Supplementary file 1 — Supplementary Information [file 41598_2018_22973_MOESM1_ESM.pdf]

Single- and two-photon imaging of human micrometastases and disseminated tumour cells with conjugates of nanobodies and quantum dots

Fernanda Ramos Gomes,[1] Julia Bode,[1] Alyona Sukhanova,[4,5] Svetlana V. Bozrova,[5] Mara Saccomano,[1] Miso Mitkovski,[2] Julia Eva Krueger,[1] Anja K. Wege,[6] Walter Stuehmer,[3] Pavel S. Samokhvalov,[5] Daniel Baty,[7] Patrick Chames,[7,*] Igor Nabiev,[4,5,*] and Frauke Alves [1,8,*]

Supporting Information Available

The videos V1 and V2 demonstrating comparison of the total emitted fluorescence at the 2P-LSM from the pAb-HER2-AF and the sdAb-HER2-QDs in deep tissue, demonstrating much stronger fluorescence emitted by the sdAb-HER2-QDs than the one emitted by the pAb-HER2-AF.

Supplementary Video V1. Detection of HER2 protein in 100 µm KPL-4 breast tumour section.

2P-LSM video shows the signal produced by pAb-HER2-AF in HER2-positive tumour cells in 100 µm agarose sections of breast tumour from KPL4 mouse models.

Supplementary Video V2. Detection of HER2 protein in 50 µm BT474 breast tumour section.

2P-LSM video showing the signals produced by sdAb-HER2-QD nanoprobes (*green signal*) in HER2-positive tumour cells in 50-µm agarose sections breast tumour from BT474 mouse models. Nuclear staining was performed with Hoechst (*red signal*).

Supplementary Figure S1. Western blot analysis using anti-HER2 antibody (left panel) shows the high expression of HER2 in human SKBR3 and BT474 cells used to produce the mouse breast tumour xenografts. A very low HER2 expression is shown in MDA-MB-231 cells used to obtain the HER2-negative control tumours. Staining of actin reveals comparable amounts of total protein in all samples (right panel).

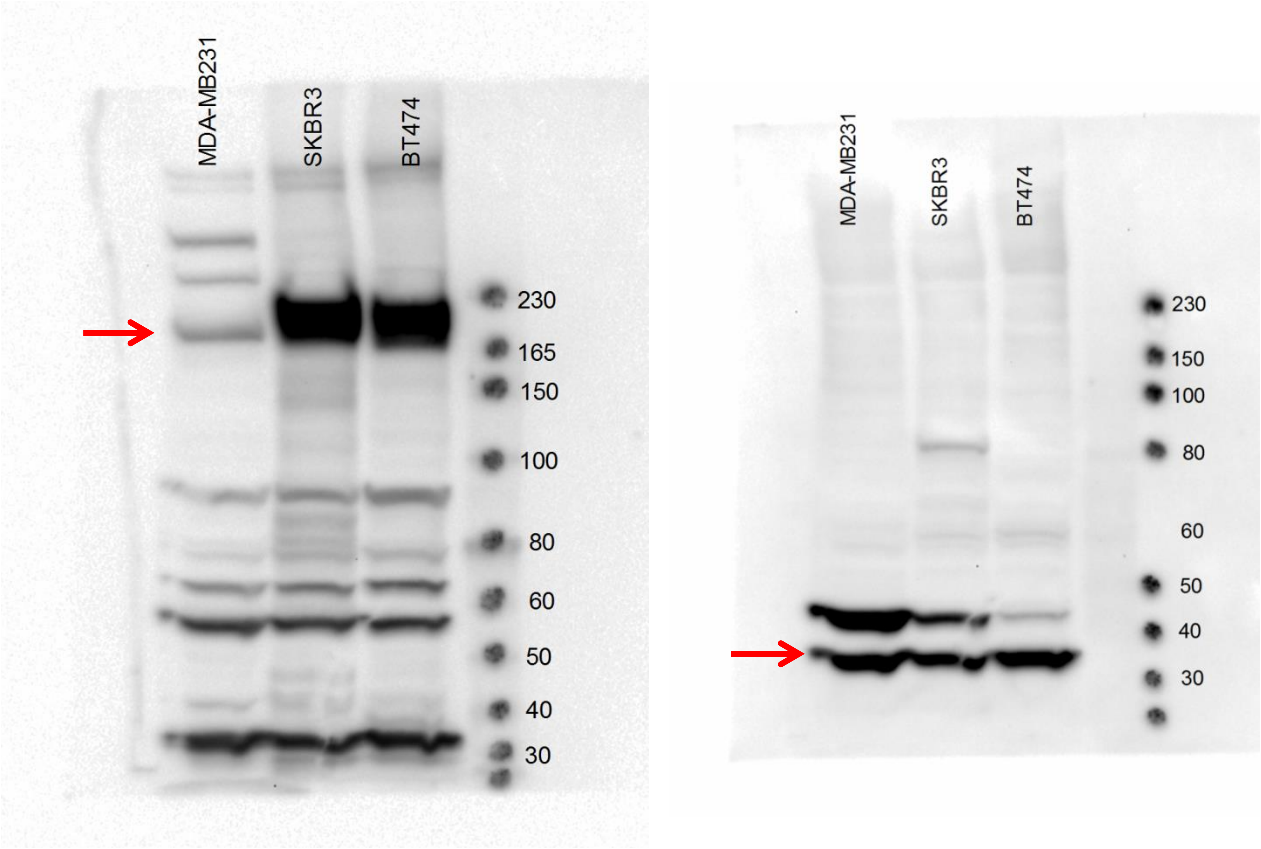

anti-HER2 antibody

anti-ß-actin antibody

Supplementary Figure S2. Western blot analysis using anti-CEA antibody (left panel) detected a high expression of CEA in AsPC1 and Capan-1 cells, which were used to produce the xenograft PDAC mouse tumours. Only low CEA expression is observed in the MIA Paca-2 cells used to obtain CEA-negative tumours as controls. Staining of actin reveals comparable amounts of total protein in all samples (right panel).

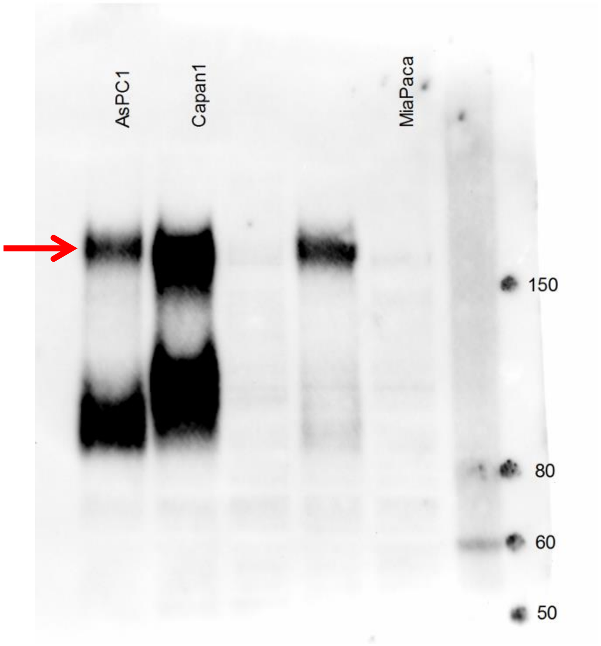

anti-CEA antibody

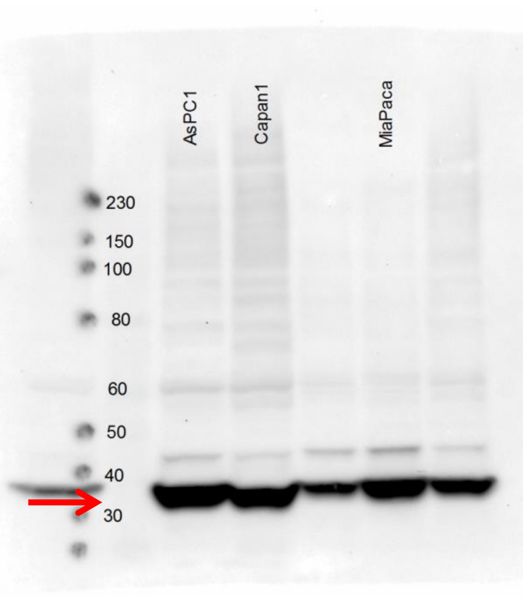

anti-ß-actin antibody
